# Long‐term evaluation and cross‐checking of two geometric calibrations of kV and MV imaging systems for Linacs

**DOI:** 10.1120/jacmp.v16i4.5140

**Published:** 2015-07-08

**Authors:** Tsuicheng D. Chiu, Yulong Yan, Ryan Foster, Weihua Mao

**Affiliations:** ^1^ Radiation Oncology Department UT Southwestern Medical Center Dallas TX USA

**Keywords:** geometric calibration, QA, kV imaging, MV imaging, image guidance, EPID

## Abstract

Geometric or mechanical accuracy of kV and MV imaging systems of two Varian TrueBeam linacs have been monitored by two geomertirc calibration systems, Varian IsoCal geometric calibration system and home‐developed gQA system. Results of both systems are cross‐checked and the long‐term geometric stabilities of linacs are evaluated. Two geometric calibration methodologies have been used to assess kV and MV imaging systems and their coincidence periodically on two TrueBeam linacs for about one year. Both systems analyze kV or MV projection images of special designed phantoms to retrieve geometric parameters of the imaging systems. The isocenters — laser isocenter and centers of rotations of kV imager and EPID — are then calculated, based on results of multiple projections from different angles. Long‐term calibration results from both systems are compared for cross‐checking. There are 24 sessions of side‐by‐side calibrations performed by both systems on two TrueBeam linacs. All the disagreements of isocenters between two calibrations systems are less than 1 mm with ± 0.1 mm SD. Most of the large disagreements occurred in vertical direction (AP direction), with an averaged disagreement of 0.45 mm. The average disagreements of isocenters are 0.09 mm in other directions. Additional to long‐term calibration monitoring, for the accuracy test, special tests were performed by misaligning QA phantoms on purpose (5 mm away from setup isocenter in AP, SI, and lateral directions) to test the liability performance of both systems with the known deviations. The errors are within 0.5 mm. Both geometric calibration systems, IsoCal and gQA, are capable of detecting geometric deviations of kV and MV imaging systems of linacs. The long‐term evaluation also shows that the deviations of geometric parameters and the geometric accuracies of both linacs are small and very consistent during the one‐year study period.

PACS number: 87.56.Fc

## I. INTRODUCTION

The primary goal of radiotherapy is to maximize or localize the treatment dose into designed target volumes and minimize the unnecessary irradiation outside of desired regions to reduce the toxicity on benign tissues or organs. One of the major components to keep us away from the goal is setup error which could come from patient positioning, machine uncertainty, revealed internal organ motion, and deformation.^1^, ^2^, ^3^ One of the best solutions is image‐guided radiation therapy (IGRT). Linear accelerators with integrated kilovoltage (kV) imaging have become routine clinical tools for IGRT and high precision of treatment is expected. It is critical to evaluate geometric accuracy and coincidence of kV imaging systems,^4^, ^5^ and also the megavoltage (MV) treatment beam, which are the baseline for limit machine uncertainty. In this study, two geometric calibration methodologies have been used to assess kV and MV cone‐beam imaging systems and their coincidence on two linear accelerators during one year.

## II. MATERIALS AND METHODS

One geometric QA system is IsoCal from Varian Medical Systems^(6)^ (Palo Alto, CA) and the other is the home‐developed gQA methodology.^(7)^ Both systems use specially designed phantoms with known ball bearings embedded — cylindrical IsoCal phantom and cuboid gQA phantom. Both systems acquire multiple kV and MV images and analyze locations of BB projections to obtain geometric parameters. The procedure of IsoCal geometric calibration is described in the handbook^(6)^ and automated procedure has been integrated into treatment console. The gQA calibration methodology has been previous reported by Mao et al.^8^, ^9^ Briefly, a phantom, gQA‐13, is designed and manufactured for geometric calibration. It is a stand‐alone solid cubic phantom containing 13 stainless steel ball bearings (BBs) mounted within a polystyrene block ($170\times 170\times 240\;{\text{mm}}^{3}$). It only requires one‐time setup without additional accessories for both kV and MV imaging system. Furthermore, it is a linac‐exchangeable algorithm/phantom and it can be easily adapted on different linacs integrating with kV and MV imaging systems. All BBs have a diameter of 4.76 mm and their 3D locations are precisely designated so that each can be easily and uniquely indentified in any projection images. The phantom has a bubble level on the top and three adjustable legs. Crosshairs are indicated to assist in phantom positioning. The phantom is initially aligned to room lasers, and MV and kV projection images are subsequently acquired at a series of gantry angles.

There are two coordinate systems used in the study. First, 3D Cartesian system (x, y, z), is used to describe the “room coordinates”. This coordinate system is attached to the phantom, which is aligned using the room lasers. The z‐axis is along the gantry rotation axis, the x‐axis is at the horizontal direction, and the y‐axis is at the vertical direction. The origin is defined by the intersection of the room lasers. Secondly, a 2D Cartesian coordinate system (u, v), which rotates with the gantry, is defined for each detector. The origin is defined at the center of the detector. The u‐axis is always in the gantry rotation plane while the v‐axis is along the gantry rotation axis.

From a single projection of the gQA‐13 phantom at a given gantry angle, nine geometric parameters are quantitatively evaluated using an identical calculation. They are the deviation of the gantry angle (ΔGA or Δφ), the deviation of SAD (ΔR), the deviation from the rotation plane (Δz), the deviation of the SDD (ΔF), the translational offsets (Δu and Δv), and the detector angular orientation (α,β,γ).

**Figure 1:**
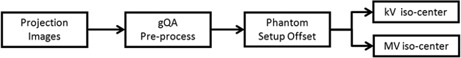
Flowchart of a complete gQA procedure.

Once each parameter is determined, isocenters are calculated based on 3D positions of the X‐ray source at multiple gantry angles during a coplanar rotation. However, all calculations are based on a coordinate system originated at the center of the gQA phantom, which is positioned manually based on laser alignment. The coincidence between gQA phantom center and interception of laser alignment is limited to the accuracy of phantom positioning. Figure 1 illustrates a completed flowchart to obtained different deviations. Details are described below.

Calculating the center of gQA phantom is based on the laser alignment. Due to the widths of the laser and the grooves on the phantom surface, the accuracy is limited to ± 0.5 mm. However, the setup error could also be caused by misaligned room lasers. If this is the scenario, the offset can be larger than 1 mm. This offset will lead to a systematic shift of both kV and MV projection centers. By applying curve fitting technique on Δu results, we can estimate the offset values in X direction and Y direction, and the offset in Z direction is corresponded to Δv results. The phantom center offsets are applied by shifting the phantom center off the origin of the coordinate system while the preprocess assumes their coincidence. The second analysis is applied to calculate all nine parameters again for every projection image. Finally, isocenters of kV and MV X‐ray systems are calculated by determining the rotation center of the X‐ray source positions based upon the second gQA analysis results.

## III. RESULTS & DISCUSSION

To access the accuracy and ability of detecting setup error, we intentionally positioned the phantoms (both gQA and IsoCal phantoms) by 5 mm offset in three orthogonal directions. Table 1 compares the results between IsoCal and gQA. Both systems could catch the shift and give good corrections. The difference of phantom setup error should be nonapplicable due to the fact that IsoCal and gQA are two individual setups and certainly they do not share the same setup errors. In the results presented in the study, the setup error corrections were applied before other parameters were processed.

**Figure 2:**
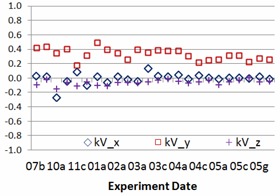
kV source rotation center offset difference (mm) between IsoCal and gQA.

**Figure 3:**
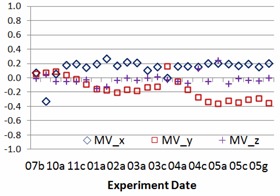
MV source rotation center offset difference (mm) between IsoCal and gQA.

The study results include 24 sessions of side‐by‐side comparisons between IsoCal and gQA procedures for each TrueBeam linacs (TB1 and TB2) from July 2013 to May 2014. The experiment frequency was intentionally increased from once a month, twice a month, once a week, twice a week, to everyday monitoring. Nine geometric parameters stated above, setup errors and centers of rotations of kV imager, and EPID are recorded in the study. Fig. 2 and Fig. 3 show center offset differences of kV imager and EPID for one of two TrueBeam linacs between IsoCal and gQA. The experience dates showing in 2, 3, 4, 5 are expressed as numbers indicating months and alphabets showing the experience orders in that specific months (i.e., 03c=the third experience in March). The results vary over the period of the past year. The average differences for kV imager center offsets are −0.01 mm (X direction), 0.34 mm (Y direction), and −0.06 mm (Z direction). The average differences for EPID center offsets are 0.14 mm (X direction), −0.15 mm (Y direction), and −0.03 mm (Z direction). Although there are center offsets between two imaging devices, the differences are in 0.01 mm scale, especially for the X and Z directions. Taking into account the mechanism of how kV imager and EPID work, the most intensive motions for both kV and EPID imager arms are happened along Y direction. It might explain larger variation along this specific axis. 4, 5 show the relative center offset between kV imager and EPID from both gQA and IsoCal results. The average relative center offsets from gQA are −0.14 mm (X direction), 0.16 mm (Y direction), and 0.02 mm (Z direction). They are 0.00 mm (X direction), −0.33 mm (Y direction), and 0.06 mm (Z direction) from the IsoCal system.

**Table 1:** TB1 and TB2 rotation center offsets comparison (mm) with (5 mm, 5 mm, 5 mm) intentional shifts

		*5 mm Phantom Setup Error*			*kV Center Offsets (mm)*			*MV Center Offset (mm)*		
		*X*	*Y*	*Z*	*X*	*Y*	*Z*	*X*	*Y*	*Z*
TB1	IsoCal	−5.92	−5.15	5.77	0.02	0.75	0.04	0.03	0.14	0.02
	gQA	−4.70	−5.80	5.70	0.12	−0.20	0.08	0.18	0.00	0.02
	Difference	N/A	N/A	N/A	−0.10	0.95	−0.04	−0.15	0.14	0.00
TB2	IsoCal	−6.35	−4.99	6.17	0.00	−0.23	−0.09	0.00	−0.08	−0.02
	gQA	−5.00	−5.20	6.20	0.06	−0.02	0.02	0.04	−0.02	0.00
	Difference	N/A	N/A	N/A	−0.06	−0.21	−0.11	−0.04	−0.06	−0.02

**Figure 4:**
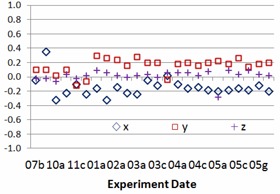
Relative distance between gQA kV and MV rotation center offsets.

By monitoring the imaging systems performance every two to three months, the results should let physicists know if the machine is getting too close to the tolerance edge. The calibration is not necessary, depending upon whether the monitored results are out of tolerance.

**Figure 5:**
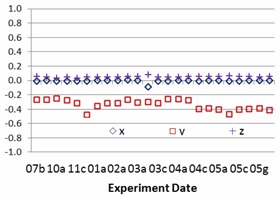
Relative distance between IsoCal kV and MV rotation center offsets.

## IV. CONCLUSIONS

Both geometric calibration systems, IsoCal and gQA, are capable of detecting geometric deviations of kV and MV imaging systems of linacs. The disagreements between two geometric calibration systems are smaller than 1 mm. Due to small and negligible differences, the study suggests two systems reveal similar geometric resultants and agree with each other. The best benefits from performing the gQA procedure is not only that we can get the most of the geometric parameters such as X‐ray source position, detector distance, kV imager and EPID center offsets, and detector orientations, but also that we can get assessments independently for phantom setup errors, and kV imager and EPID center offsets from the same procedure. The long‐term evaluation also shows that the geometric accuracy is very consistent over the period of about one year. The study results provide evidences of TrueBeam reliability by being confirmed by both Varian IsoCal and our gQA procedures.

## ACKNOWLEDGMENTS

This work was supported by a grant from Varian Medical System.

## Supporting information

Supplementary Material

Supplementary Material
