# Supplementary figures and images for: Long‐term evaluation and cross‐checking of two geometric calibrations of kV and MV imaging systems for Linacs
Source: J Appl Clin Med Phys. 2015 Jul 8;16(4):306–10. doi: 10.1120/jacmp.v16i4.5140 (PMC5690018; doi:10.1120/jacmp.v16i4.5140)

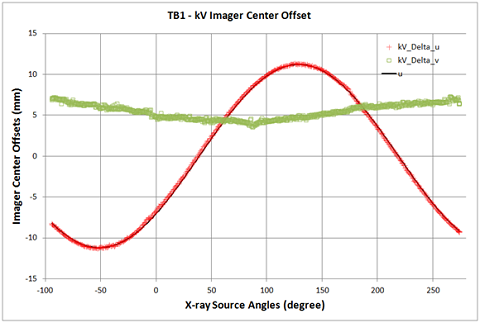

Supplement: Supplementary file 1 — Supplementary Material [file ACM2-16-306-s001.png]

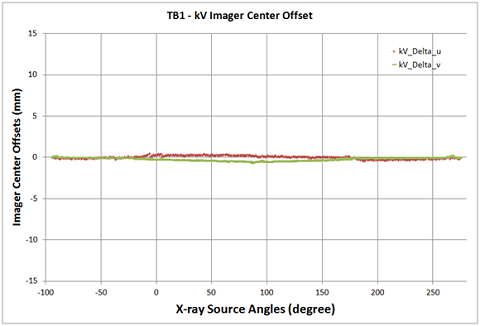

Supplement: Supplementary file 2 — Supplementary Material [file ACM2-16-306-s002.png]
